# Conservation of Carbohydrate Binding Interfaces — Evidence of Human HBGA Selection in Norovirus Evolution

**DOI:** 10.1371/journal.pone.0005058

**Published:** 2009-04-01

**Authors:** Ming Tan, Ming Xia, Yutao Chen, Weiming Bu, Rashmi S. Hegde, Jarek Meller, Xuemei Li, Xi Jiang

**Affiliations:** 1 Division of Infectious Diseases, Cincinnati Children's Hospital Medical Center, Cincinnati, Ohio, United States of America; 2 Department of Pediatrics, University of Cincinnati College of Medicine, Cincinnati, Ohio, United States of America; 3 Division of Developmental Biology, Cincinnati Children's Hospital Medical Center, Cincinnati, Ohio, United States of America; 4 Department of Environmental Health, University of Cincinnati College of Medicine, Cincinnati, Ohio, United States of America; 5 National Laboratory of Bio macromolecules, Institute of Biophysics, Chinese Academy of Science, Beijing, China; University of Minnesota, United States of America

## Abstract

**Background:**

Human noroviruses are the major viral pathogens of epidemic acute gastroenteritis. These genetically diverse viruses comprise two major genogroups (GI and GII) and approximately 30 genotypes. Noroviruses recognize human histo-blood group antigens (HBGAs) in a diverse, strain-specific manner. Recently the crystal structures of the HBGA-binding interfaces of the GI Norwalk virus and the GII VA387 have been determined, which allows us to examine the genetic and structural relationships of the HBGA-binding interfaces of noroviruses with variable HBGA-binding patterns. Our hypothesis is that, if HBGAs are the viral receptors necessary for norovirus infection and spread, their binding interfaces should be under a selection pressure in the evolution of noroviruses.

**Methods and Findings:**

Structural comparison of the HBGA-binding interfaces of the two noroviruses has revealed shared features but significant differences in the location, sequence composition, and HBGA-binding modes. On the other hand, the primary sequences of the HBGA-binding interfaces are highly conserved among strains within each genogroup. The roles of critical residues within the binding sites have been verified by site-directed mutagenesis followed by functional analysis of strains with variable HBGA-binding patterns.

**Conclusions and Significance:**

Our data indicate that the human HBGAs are an important factor in norovirus evolution. Each of the two major genogroups represents an evolutionary lineage characterized by distinct genetic traits. Functional convergence of strains with the same HBGA targets subsequently resulted in acquisition of analogous HBGA binding interfaces in the two genogroups that share an overall structural similarity, despite their distinct locations and amino acid compositions. On the other hand, divergent evolution may have contributed to the observed overall differences between and within the two lineages. Thus, both divergent and convergent evolution, as well as the polymorphic human HBGAs, likely contribute to the diversity of noroviruses. The finding of genogroup-specific conservation of HBGA binding interfaces will facilitate the development of rational strategies to control and prevent norovirus-associated gastroenteritis.

## Introduction

Noroviruses, a group of single-stranded, positive sense RNA viruses, constitute one of the six genera of the family *Caliciviridae* [1], [2], [3]. Noroviruses are genetically highly diverse containing 5 genogroups (G) and ∼30 genotypes [4]. GI, GII and GIV infect humans and cause acute gastroenteritis [1], [5], [6], GIII infect cattle causing similar diseases [7], while GV infect mice and cause disease only in immune compromised mice [8]. GI and GII are studied extensively owing to their importance in human disease. Although some genogroup-specific features related to the epidemiology and environmental transmission have been analyzed [9], [10], [11], the biological significance and evolutionary relationships regarding the HBGA interaction of the two major genogroups of human noroviruses requires further elucidation.

Noroviruses contain a major structural protein (VP1) that forms the viral capsid [12]. The VP1 has two principle domains, the shell (S) and the protruding (P) domains, linked by a short hinge. The S domain forms the interior icosahedral shell, while the P domain constitutes the arch-like P dimer protruding from the shell. The P domain can be further divided into two subdomains, P1 and P2, with the P2 subdomain at the outermost surface of the viral capsid [12], [13], [14], [15]. The S and P domains appear to be structurally and functionally independent, as suggested by the facts that the P domain alone forms a dimer and P particle (12 P dimers), and both P dimer and P particle retain receptor-binding function [16], [17], [18], while the S domain alone forms S particle without receptor-binding function [16]. In addition, a large amount of soluble P protein has been found in the stools of Norwalk virus-infected patients [18], [19], [20], although its biological significance remains unknown.

Human noroviruses recognize human histo-blood group antigens (HBGAs), most likely as receptors or co-receptors ([21], [22], [23], [24], [25], [26], [27], [28], [29], reviewed in [30], [31]). HBGAs are complex carbohydrates linked to proteins or lipids on the surface of red blood cells and mucosal epithelia of the respiratory, genitourinary, and digestive tracts, or as free oligosaccharide in biological fluids such as milk and saliva. A number of distinct binding patterns of noroviruses to HBGAs have been described according to the ABO, Lewis and secretor types of the human HBGAs [21], [22], [32]. The prototype Norwalk virus (GI-1) represents one of these patterns and binds to saliva of A and O secretors. Other binding patterns include the A, B, O secretor binder of VA387 (GII-4), A, B binder of MOH (GII-5), and A, O secretor and nonsecretor binder of VA207 (GII-9) and Boxer (GI-8) [22]. The variable binding patterns have been further sorted into two major binding groups, the A/B binding group and Lewis binding group based on shared HBGA targets within binding groups. The A/B binding group recognizes mainly the A/B/H but not the Lewis epitopes, while the Lewis binding group binds the Lewis epitopes but not the A/B epitopes ([22], reviewed in [30], [31]). Strains of both A/B and Lewis binding groups can be found in the two major genogroups of human noroviruses. In addition, virus-HBGA interaction has also been found in two other genera of caliciviruses. For example, the rabbit hemorrhagic disease virus (RHDV) binds to H antigen [33], while the Tulane virus binds to B antigen [34], suggesting that the involvement of HBGAs in calicivirus infection may be a common phenomenon.

**Figure 1:**
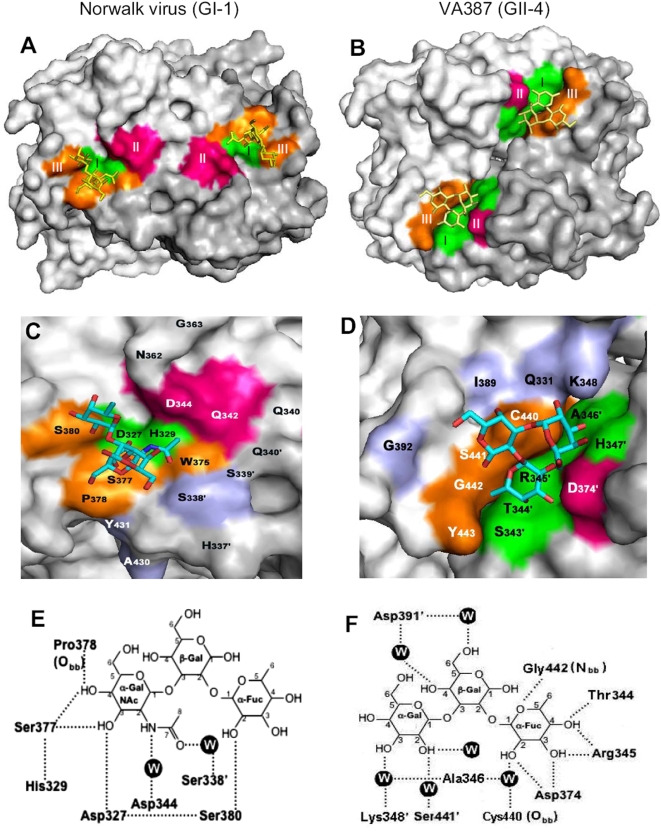
The crystal structures of the HBGA-binding interfaces of Norwalk virus (GI-1) and VA387 (GII-4). The surface models of the P dimers (top views) with indications of the HBGA-binding interfaces (colored regions) are shown in (A) and (B) with one monomer being shown in darker gray than another. Enlargements of the HBGA-binding interfaces are shown in (C) and (D) correspondingly with labels of individual amino acids, in which the prime symbol indicates a residue of another protomer. The three major components of the binding interfaces are colored in green (site I), red (site II), and orange (site III), respectively, while the trisaccharides binding to the interface are in yellow in (A) and (B) or in variable colors (C-cyans, O-red, and N-blue) in (C) and (D). The amino acids around the interface that affect the binding specificity are in light blue. (E) and (F) are schematic diagrams of hydrogen bonding network (dash lines) between the amino acids of the P dimers of Norwalk virus (E), or VA387 (F) and the A- or B- type trisaccharides. The water-bridged hydrogen bonds are indicated by W. (A) to (D) were prepared by software PyMOL version 1.0 (Delano Scientific), while (E) and (F) by software ChemDraw Pro version 11.0 (Adept Scientific). (E) is adapted from [13] with permission. The original data were published in [13], [14], [15].

High resolution 3D structures of HBGA-binding interfaces of Norwalk virus (GI-1) and VA387 (GII-4) have recently been determined [13], [14], [15]. As shown by these studies, the binding interfaces of both strains are located in the same region of the outermost P2 domain, but the positions and amino acid composition of individual binding sites and the HBGA binding modes are different. The HBGA-binding interface of Norwalk virus is located within a single monomer of the P dimer (Fig. 1, left panel), while the binding interface of VA387 involves both monomers of the P dimer (Fig. 1, right panel), although in both cases dimerization of the P domains appears to be required for their binding function [13], [14], [35]. While both Norwalk virus and VA387 belong to the A/B binding group and share the same A and H antigens, the primary sequences of their binding sites and the binding modes to HBGAs are different. Norwalk virus interacts with the α-GalNAc and α-Fus of the A trisaccharide or α-Fuc and β-Gal of the H tetrasaccharide [13], [15], while VA387 interacts with all three terminal sugars of A (α-GalNAc-β-Gal-α-Fuc) and B (α-Gal-β-Gal-α-Fuc) trisaccharides [14].

In this study, we characterized the genetic relatedness of human noroviruses in the context of their host carbohydrate-binding specificity by sequence alignment and structural analysis, followed by site-directed mutagenesis and functional assay for strains representing both GI and GII and both A/B and Lewis binding groups. We showed that the HBGA-binding interfaces are highly conserved among strains within, but not between, the two genogroups, and that the conserved binding interfaces are able to interact with variable HBGAs of the ABH and Lewis families. Our results suggested that human HBGA may be a selection factor in norovirus evolution. The polymorphic human HBGAs and the highly adaptative nature of noroviruses may underlie the observed diversity of noroviruses. The high conservation of HBGA-binding interfaces within genogroups may also help in the development of new strategies to control and prevent norovirus infection.

## Materials and Methods

### Construction of mutant P particles

Bacterial expression constructs for wild type P particles were made by cloning of the P domain-encoding sequences into the plasmid pGEX-4T-1 (Amersham Bioscience, Piscataway, NJ). To enhance the efficiency of P particle formation a cysteine-containing short peptide was linked to either the N- (Norwalk virus, CNGRC) or C- (Boxer, MOH, and VA207, CDCRGDCFC) termini of the P domains, as reported previously [16], [17], [18], [35], [36]. Mutant P particles with single amino acid substitutions were designed and constructed by site-directed mutagenesis using the corresponding wild type constructs as templates. Site-directed mutagenesis was performed using the QuickChange Site-Directed Mutagenesis Kit (Stratagene, La Jolla, CA) and the corresponding primer pairs with mutations (Table 1) as described previously [16], [17], [18], [35], [36]. The wild type and mutant P particles were expressed and purified as described previously [16], [17], [18], [35], [36]. Briefly, after sequence confirmation through DNA sequencing, the mutant constructs were expressed in *E. coli* strain BL21 with an induction of IPTG (0.25 mM) at room temperature (∼25°C) overnight. The P protein-GST fusion was purified by the Glutathione Sepharose 4 flow (GE Healthcare Life Sciences, Piscataway, NJ) according to the manufacturer's protocol. P proteins were released from GST tag by thrombin (GE Healthcare Life Sciences, Piscataway, NJ) digestion. The formation of P particle and P dimer was determined by gel filtration using a size-exclusion column Superdex 200 (GE Healthcare Life Sciences, Piscataway, NJ) powered by an AKTA-FPLC system (model 920, GE Healthcare Life Sciences, Piscataway, NJ) followed by SDS-PAGE electrophoresis, in which the P particles form a peak at ∼830 kDa and the P dimer at ∼69 kDa, respectively previously [16], [17], [18], [35], [36]. The efficiency of P particle formation for Norwalk virus, Boxer, MOH, and VA207 were ∼70%, ∼80%, ∼80%, and ∼90%, respectively. None of the designed single residue mutations in this study affected P particle formation.

**Table 1:** Primers used for cloning of P domain and site-directed mutagenesis to generate single mutation in the HBGA-binding interface and around regions.

Name	Primer sequence (5′ to 3′)	Sense	Mutation
	Primers for Norwalk virus (GI-1)		
P588	gcgtggatcctgcaacggccgttgctttttagtccctcctacggtg	+	Wild type
P494	ggacgcggccgcttatcggcgcagaccaagcct	−	Wild type
P1227	cctcggtggttgtgcttggcatatcaatatg	+	D327A
P1228	catattgatatgccaagcacaaccaccgagg	−	D327A
P1122	ggtggttgtgattgggctatcaatatgacacag	+	H329A
P1123	ctgtgtcatattgatagcccaatcacaaccacc	−	H329A
P1124	atgacacagtttggcgcttctagccagacccag	+	H337A
P1125	ctgggtctggctagaagcgccaaactgtgtcatattg	−	H337A
P1126	acacagtttggccatgctagccagacccagtatg	+	S338A
P1127	atactgggtctggctagcatggccaaactgtgtc	−	S338A
P1271	acacagtttggccataatagccagacccagtatg	+	S338N
P1272	cacactgggtctggctattatggccaaactgtgt	−	S338N
P1128	cagtttggccattctgcccagacccagtatgatg	+	S339A
P1129	atcatactgggtctgggcagaatggccaaactgtg	−	S339A
P1231	gcatatcaatatgacagcgacccagtatgatg	+	Q340A
P1232	catcatactgggtcgctgtcatattgatatgc	−	Q340A
P1130	cattctagccagaccgcgtatgatgtagacaccacc	+	Q342A
P1131	ggtgtctacatcatacgcggtctggctagaatggc	−	Q342A
P1255	cagacccagtatgvtgtagacaccacc	+	D344A
P1256	ggtggtgtctacagcatactgggtctg	−	D344A
P1132	ggttcaattcaggcagctggcattggcagtgg	+	N362A
P1133	accactgccaatgccagctgcctgaattgaacc	−	N362A
P1134	ggttcaattcaggcaaatgccattggcagtggtaattatg	+	G363A
P1135	attaccactgccaatggcatttgcctgaattgaacc	−	G363A
P1136	gttggtgttcttagcgcgatttcccccccatcac	+	W375A
P1137	tgatgggggggaaatcgcgctaagaacaccaac	−	W375A
P1240	gttcttagctggattgcccccccatcacac	+	S377A
P1239	gtgtgatgggggggcaatccagctaagaac	−	S377A
P1241	cttagctggatttccgccccatcacacccg	+	P378A
P1242	cgggtgtgatggggcggaaatccagctaag	−	P378A
P1243	ggatttcccccccagcacacccgtctggc	+	S380A
P1244	gccagacgggtgtgctgggggggaaatcc	−	S380A
P1138	atgccaggtcctggtacttataatttgccctgtc	+	A430S
P1139	gacagggcaaattataagtaccaggacctggc	−	A430S
P1140	ccaggtcctggtgctgctaatttgccctgtctattacc	+	Y431A
P1141	tagacagggcaaattagcagcaccaggacctgg	−	Y431A
	Primers for Boxer (GI-8)		
P744	cgcggatcccaaagaaccaagccatttagtg	+	Wild type
P746	ataagaatgcggccgcttagcaaaagctaactgccacggcaatcgca		
	tgatctcctgagaccaagcct	−	Wild type
P1011	ggaaattgtgatttggctatgacctttgttaag	+	H334A
P1012	cttaacaaaggtcatagccaaatcacaatttcc	−	H334A
P1042	catatgacctttgttaaggctaatcccactgagttgtcc	+	I340A
P1043	caactcagtgggattagccttaacaaaggtcatatgc	−	I340A
P1013	gacctttgttaagattgctcccactgagttgtcc	+	N341A
P1014	ggacaactcagtgggagcaatcttaacaaaggtc	−	N341A
P1044	cctttgttaagattaatgctactgagttgtccactg	+	P342A
P1045	cagtggacaaatcagtagcattaatcttaacaaagg	−	P342A
P1046	ccactgagttgtccgctggagatccttctggtaag	+	T347A
P1047	accagaaggatctccagcggacaactcagtggg	−	T347A
P1015	cactgagttgtccactgcagatccttctggtaag	+	G348A
P1016	cttaccagaaggatctgcagtggacaactcagtg	−	G348A
P1048	gagttgtccactggagctccttctggtaaggtg	+	D349A
P1049	caccttaccagaaggagctccagtggacaactc	−	D349A
P1017	gttgtccactggagatgcttctggtaaggtggtc	+	P350A
P1018	gaccaccttaccagaagcatctccagtggacaac	−	P350A
P1050	ctggaggataataatgctttagatcagtttgtgg	+	E377A
P1051	cacaaactgatctaaagcattattatcctccag	−	E377A
P1052	gaggataataatgaggctgatcagtttgtgggc	+	L378A
P1053	ccacaaactgatcagcctcattattatcctcc	−	L378A
P1019	gaggataataatgagttagctcagtttgtgggcaag	+	D379A
P1020	cttgcccacaaactgagctaactcattattatcctc	−	D379A
P1054	gataataatgagttagatgcttttgtgggcaaggaag	+	Q380A
P1055	ttccttgcccacaaaagcatctaactcattattatcctc	−	Q380A
P1056	aatgagttagatcaggctgtgggcaaggaagtgg	+	F381A
P1057	cacttccttgcccacagcctgatctaactcattattatcc	−	F381A
P1021	gtgctggagatgacggcggtttccaatagaacgg	+	W392A
P1022	cgttctattggaaaccgccgtcagctccagcag	−	W392A
P1058	catttccaacggtcagtgctccaaaagttccatgtacc	+	N444A
P1059	gtacatggaacttttggagcactgaccgttggaaatgtgg	−	N444A
	MOH (GII-5)		
P1309	cgcggatcctcaaagactaagccatttacac	+	Wild type
P1310	ataagaatgcggccgctaagcaaaagcaatctogccacggcaatcgca		
	ctgaaaccttctgcgcccattc	−	Wild type
P1344	gcaacccagcaaacgcggctcatgatgctg	+	R347A
P1345	cagcatcatgagccgcgtttgctgggttgc	−	R347A
P1346	cttggaacaccaatgctgttgaaaaccaacc	+	D376A
P1347	ggttggttttcaacagcattggtgttccaag	−	D376A
P1348	cccattaaaaggtgcatttggaaaccctg	+	G441A
P1349	cagggtttccaaatgcaccttttaatggg	−	G441A
	VA207 (GII-9)		
P709	acgcgtcgactctcaaagactaaggcattcac	+	Wild type
P702	gcgtgcggccgcttagcaaaagcaatcgccatggcaatcgcattgg		
	atccttctccccccacttcc	−	Wild type
P1338	caggtgacgccacggcggcccatgaggcaag	+	R346A
P1339	cttgcctcatgggccgccgtggcgtcacctg	−	R346A
P1340	ctcaacctcaagcgcttttgaaacaaacc	+	D374A
P1341	ggtttgtttcaaaagcgcttgaggttgag	−	D374A
P1342	ccaggagctagtgcccacacaaatggg	+	G440A
P1343	cccatttgtgtgggcactagctcctgg	−	G440A

### Saliva-based HBGA binding assay

These were performed as described elsewhere [21], [22], [35], [36]. The affinity-column purified P particles were first diluted to 1 mg/ml as a starting solution. They were then diluted further in a 3-fold-series to indicated concentration directly on the testing Elisa plates that had been coated with different saliva. The different P particles were incubated with coated saliva samples for 60 min at 37°C. Five well-characterized saliva samples representing typical blood types of “O”, “A”, “B”, “AB” secretor and “O” nonsecretor [21], [22] were used for the binding assays.

### Crystal structure visualization and analysis

The crystal structures of the P dimers of Norwalk virus and VA387 complexed with type A-, B-trisaccharides, and/or H-pentasaccharide were analyzed using the PyMOL software (DeLano Scientific LLC, Palo Alto, CA) and the Polyview-3D server (http://polyview.cchmc.org). The PDB files of the Norwalk virus P protein in complex with A-trisaccharide (3D26 and 2ZL7) or H-pentasaccharide (2ZL6) [13], [15] and VA387 P protein in complex with A-trisaccharide (2OBS) or with B-trisaccharide (2OBT) [14] were downloaded from the Protein Data Bank at Rutgers University, New Brunswick, NJ (http://www.rcsb.org).

## Results

### Characterization of the HBGA-binding interface of Norwalk virus

The prototype Norwalk virus (GI-1) and strain VA387 (GII-4), each representing genogroups (G) I and II of human noroviruses, exhibit distinct HBGA binding patterns but share the ability to bind to the A and H antigens [22]. Crystallographic studies indicated that these two strains use distinct binding interfaces and modes of interaction with HBGA receptors (Fig. 1, [13], [14], [15]). Our recent site-directed mutagenesis analysis of VA387 has identified a number of additional amino acids around the carbohydrate binding interface that are also involved in HBGA binding [35]. To further elucidate the differences and similarities between the two HBGA-binding interfaces and modes, we extended such mutagenesis analysis to the Norwalk virus.

**Figure 2:**
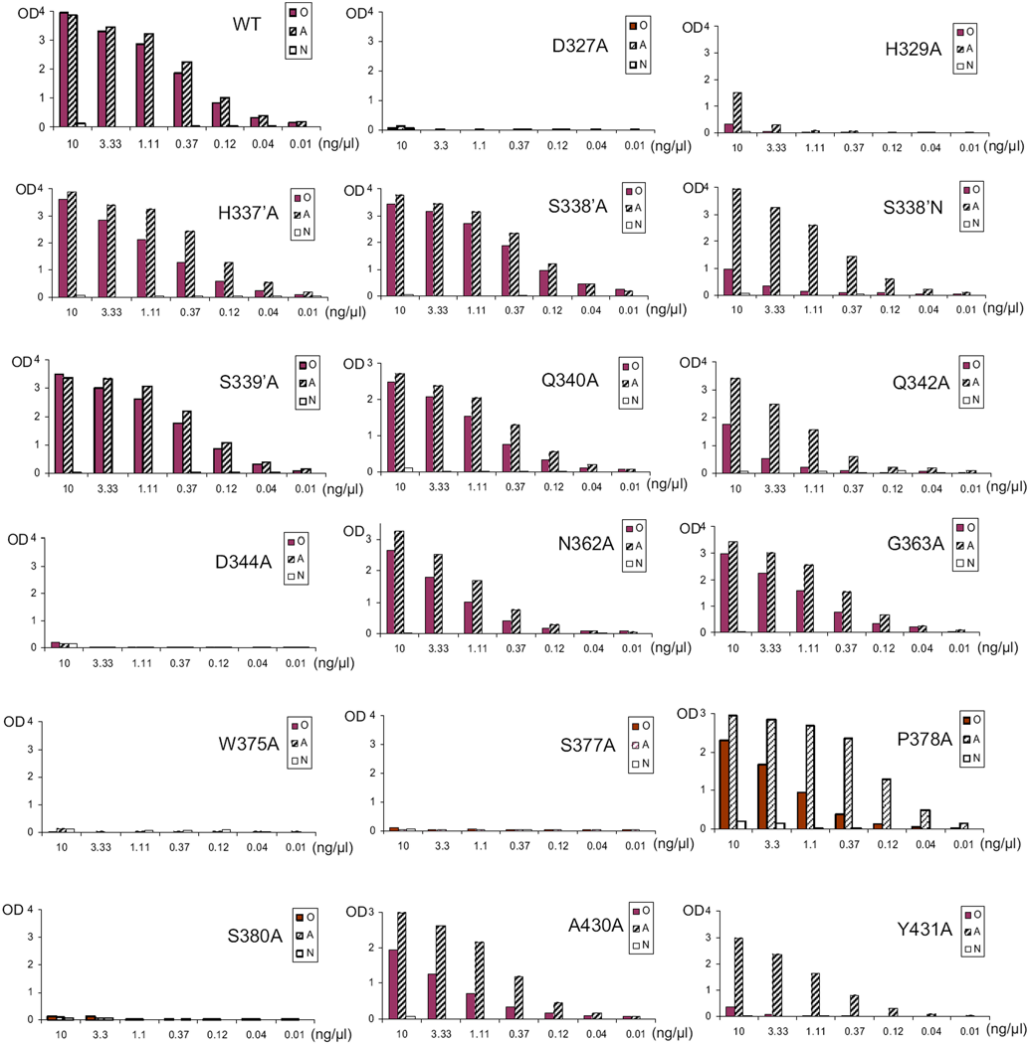
Binding of various mutant P particles with single amino acid changes in or around the HBGA-binding interface of Norwalk virus to the saliva samples. X-axes show protein concentrations of the P particles and Y axes indicate the optical densities at 450 nm (OD_450_) that were the average values of triplicate experiments. “O”, “A” and “N” represent the saliva samples of type O secretor (containing H antigen), type A secretor, and type O nonsecretor, respectively. Data of mutations D327A, H329A, S338'A, and S377A are adapted with permission from [13]. Mutants with prime symbol (') indicate the mutated residues of another P monomer.

Among 17 mutant P particles with single amino acid changes into alanines/serines in and around the HBGA-binding interface of the Norwalk virus (Fig. 2 and Table 2), six mutants (D_327_A, H_329_A, D_344_A, W_375_A, S_377_A, and S_380_A) lost their binding to HBGAs completely or nearly completely (H_329_A) (Fig. 1 and Fig. 2, [13]), indicating that these amino acids are critical for the structural integrity of the binding interface. Residue Q_342_ that was shown to interact with the type H but not the type A oligosaccharide [13], [15] affected binding mainly to the H when it was replaced by an alanine (Fig. 2). Similar effects were also seen in mutants P_378_A, A_430_S, and Y_431_A, respectively, although P_378_ was predicted to interact with both types H and A oligosaccharides, while A_430_ and Y_431_ do not appear to interact with either of the two oligosaccharides [13], [15]. Furthermore, a replacement of S_338_ by an alanine (S_338_A) did not affect binding to either A or H antigens (Fig. 2 and [13]), but a change to an asparagine (S_338_N) wiped out binding to H without affecting binding to A antigen (Fig. 2), although S_338_ from a heterologous P monomer interacted with the α-N-acetylgalacosamine (α-GalNAc) of the A antigen via a water-bridged hydrogen bond [13]. The involvement of residues S_338_, P_378_, A_430_, and Y_431_ in HBGA-binding specificity may be supported by their common location in a region on one side of the binding interface (Fig. 1C), although the mechanism remains to be elucidated. In contrast, although residues H_337_, S_339_, Q_340_, N_362_, and G_363_ are also located around the binding interface, their mutations into alanines did not have significant impact on binding to HBGAs (Fig. 2 and Table 2).

**Table 2:** Summary of the mutagenesis study of the amino acid residues in and around the predicted HBGA-binding interface of Norwalk virus.

Mutants^1^	Binding to type O saliva^2^	Binding to type A saliva^2^	Binding to saliva of nonsecretor^2^	Predict to interact with HBGAs^3^
WT	++++	++++	−	
D_327_A	−	−	−	H and A
H_329_A	−	+	−	H and A
*H_337_A*	+++	++++	−	no
*S_338_A*	++++	++++	−	A
*S_338_N*	+	++++	−	A
*S_339_A*	++++	++++	−	no
Q_340_A	+++	+++	−	no
Q_342_A	+	+++	−	H
D_344_A	−	−	−	H and A
N_362_A	++	+++	−	no
G_363_A	+++	++++	−	no
W_375_A	−	−	−	H and A
S_377_A	−	−	−	H and A
P_378_A	++	+++	−	H and A
S_380_A	−	−	−	A
A_430_S	++	+++	−	no
Y_431_A	−	+++	−	no

1 The P particle formation of the wild type (WT) and all mutants were confirmed by gel filtration. The italicized mutants in indicate the mutated residues of another P monomer.

2 The number of “+” indicated the relative binding affinity of the wild type and mutant P particles at 3.33 ng/µl to HBGAs: “++++” indicates an OD_450_ >3.0; “+++” between 2.0 and 3.0; “++” between 1.0 and 2.0; “+” between 0.3 and 1.0; while “−” indicates an OD_450_ <0.3, suggesting a complete loss of binding. Type O and A saliva were from secretors containing H and A antigen, respectively.

3 Predictions were made by two independent crystallographic studies (ref13 and 15).

### Distinct HBGA-binding interfaces and modes between Norwalk virus and VA387

The crystal structures of the binding interfaces [13], [14], [15] and the site-directed mutagenesis ([35]and data in the previous section) suggest that the HBGA-binding interfaces of both Norwalk virus and VA387 can be divided into three major analogous regions, representing the bottom (site I) and the walls (site II and III) of the interface (Fig. 1). These sites are composed of either a single or a cluster of sterically closed amino acids, including D_327_ and H_329_ (site I), Q_342_ and D_344_ (site II), and W_375_, S_377_, and S_380_ (site III) for Norwalk virus and S_343_ to H_347_ (site I), D_374_ (site II), and S_441_ and G_442_ (site III) for VA387, but none of these sites are shared by the two strains. It should be noted that all three sites of Norwalk virus are formed by residues of a single P2 subdomain without direct interactions with the P1 subdomain or the dimer-related P2 subdomain, while site III (S_441_ and G_442_) of VA387 is formed by the top of an exposed loop of P1 subdomain, and it involves the other chain of the VA387 P dimer. In both strains these three sites interact with at least two sugars of the A, B or H antigens. However, in Norwalk virus the major contacts are on the α-N-acetyl galactosamine (α-GalNAc) of the A trisaccharide or the β-galactose (β-Gal) of H pentasaccharide [13], [15], while in VA387 the major contacts are on the α-fucose (α-Fuc) of the A and B trisaccharides [14].

### The HBGA-binding interfaces are conserved within but not between genogroups

**Figure 3:**
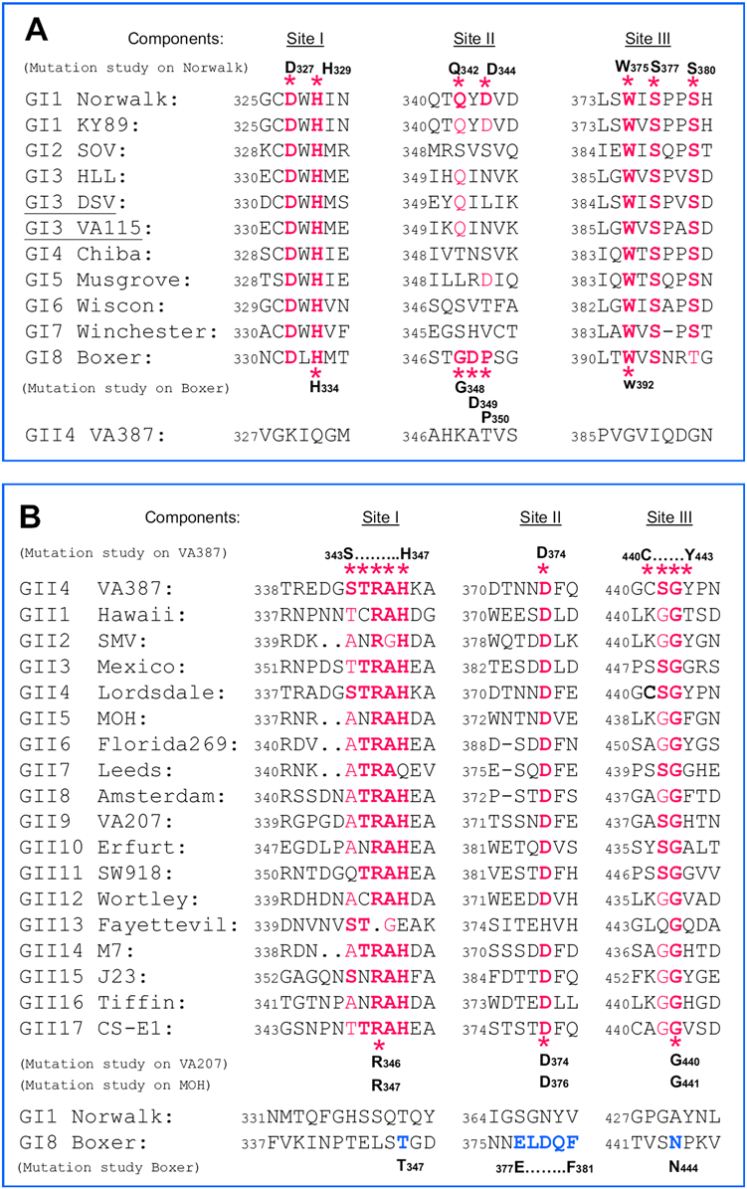
Sequence alignments of the HBGA-binding interfaces of various GI and GII noroviruses. Sequence of the three major components (red letters) of the HBGA-binding interfaces of 10 genogroup I (GI) (A) and 17 genogroup II (GII) (B) noroviruses, representing each of the 8 GI and 17 GII genetic types, respectively, are aligned based on the two known binding interfaces of Norwalk virus (GI) and VA387 (GII). Star symbols label the residues that have been experimentally shown to be required for binding to HBGAs. The two strains that have no detectable binding to examined HBGAs are underlined. The accession numbers of the sequence are: M87661 (Norwalk virus), L23828 (KY 89), L07418 (SOV), AF414403 (HLL), U04469 (DSV), AY038598 (VA115), AB042808 (Chiba), AJ277614 (Musgrove), AY502008 (Wiscon), AJ277609 (Winchester), AF538679 (Boxer), AY038600 (VA387), U07611 (Hawaii), AY134748 (SMV), U22498 (Mexico), X86557 (Lordsdale), AF397156 (MOH), AF414407 (Florida269), AJ277608 (Leeds), AF195848 (Amsterdam), AAK84676 (VA207), AF427118 (Erfurt), AB074893 (SW918), AJ277618 (Wortley), AY113106 (Fayettevil), AY130761 (M7), AY130762 (J23), AY502010 (Tiffin), AY502009 (CS-E1).

Sequence alignments of the P domains of noroviruses representing 8 GI and 17 GII genotypes (Fig. 3) show that the three sites of the HBGA-binding interfaces are highly conserved within each genogroup. Sites I and III are more conserved than site II for the GI viruses, while all three sites are highly conserved among GII viruses except for strains of GII-13, including site III that is in the P1 subdomain of the capsid (Fig. 1 and 3). The overall sequence identities of the P2 subdomains are only 31–56% for strains within each of the two genogroups, further indicating the selective pressures of the HBGAs on the receptor binding interfaces.

### All three conserved sites are required for Boxer (GII-8) binding to HBGAs

The high conservation of the HBGA-binding interfaces raises an important question on the role of human HBGAs in norovirus evolution. For strains with similar binding patterns within the same genogroups, such as Norwalk virus (GI-1) and C59 (GI-2) in the A/B binding group that both bind to types A and H antigens of secretors but not to the Lewis antigens of the non-secretors [22], such conservation is understandable. However, the conservation of HBGA binding interfaces among strains with distinct binding patterns, such as Boxer (GI-8) of the Lewis binding group [22], seems to challenge the hypothesis that HBGAs confer selective pressure on norovirus evolution.

**Figure 4:**
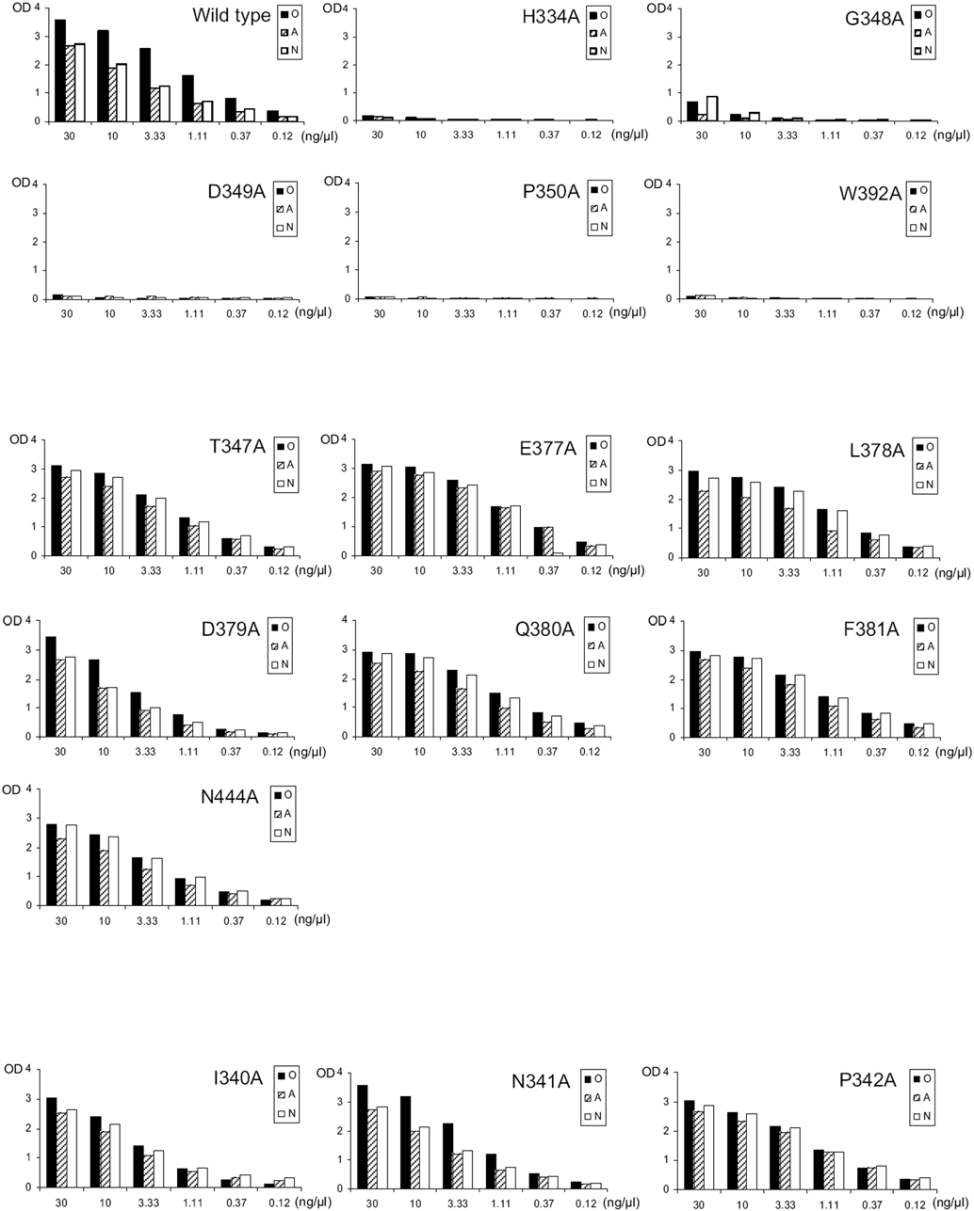
Saliva-based binding results of various mutant P particles of Boxer (GI-8) with single amino acid changes at the three GI conserved sites (upper panel), at the regions corresponding to the three GII-conserved sites (middle panel), and at regions away from the predicted binding interface (lower panel). The X-axes show the protein concentrations of the P particles and the Y axes indicate the optical densities at 450 nm (OD_450_) that were the average value of triplicate experiments. “O” and “A” represent the saliva samples of type O (containing H antigen) and A secretor, respectively, while “N” one of nonsecretor.

To address this apparent inconsistency and further elucidate the relationship between structure and HBGA binding patterns, we performed mutagenesis analysis on the role of the three conserved sites in HBGA binding of the Boxer virus. Three sets of mutant P particles were constructed. The first set contained 5 mutants with single residue mutations in each of the three GI conserved sites (Fig. 3A), including H_334_A (site I), G_348_A, D_349_A, P_350_A (site II), and W_392_A (site III); the second set were 7 mutants with mutations in regions corresponding to each of the three GII conserved sites (Fig. 3B), including T_347_A (site I), E_377_A, L_378_A, D_379_A, Q_380_A, F_381_A (site II), and N_444_A (site III); while the third set contained 3 mutants with mutations (I_340_A, N_341_A and P_342_A) away from the predicted binding interface as control (Fig. 4 and Table 3). The saliva-based binding results showed that all 5 mutants of the first set but none of the 7 mutants in the second set lost their binding to HBGAs completely or nearly completely (Fig. 4 and Table 3). As expected, the three mutants in the third set did not affect the binding. Therefore, all three conserved sites deduced from the GI Norwalk virus for the A/B binding group are also involved in HBGA-binding of the GI Boxer virus of the Lewis binding group. These data provided functional evidence for the conservation of the HBGA binding interface of GI noroviruses.

**Table 3:** Summary of the mutagenesis study of the three sites of the HBGA-binding interface of Boxer virus (GI-8) predicted by sequence alignment with Norwalk virus (GI) and VA387 (GII).

Mutants	Binding to type O saliva^1^	Binding to type A saliva^1^	Binding to saliva of nonsecretor^1^	Components of the binding interface
WT	++++	+++	+++	
H_334_A	−	−	−	GI-site I
G_348_A	−	−	+	GI-site II
D_349_A	−	−	−	GI-site II
P_350_A	−	−	−	GI-site II
W_392_A	−	−	−	GI-site III
T_347_A	++++	+++	+++	GII-site I
E_377_A	++++	+++	+++	GII-site II
L_378_A	+++	+++	+++	GII-site II
D_379_A	+++	++	++	GII-site II
Q_380_A	+++	+++	+++	GII-site II
F_381_A	+++	+++	+++	GII-site II
N_444_A	+++	++	+++	GII-site III
I_340_A	+++	+++	+++	control
N_341_A	++++	+++	+++	control
P_342_A	+++	+++	+++	control

1 The number of “+” indicated the relative binding affinity of the wild type (WT) and mutant P particles at 10 ng/µl to HBGAs: “++++” indicates an OD_450_ >3.0; “+++” between 2.0 and 3.0; “++” between 1.0 and 2.0; “+” between 0.3 and 1.0; while “−” indicates an OD_450_ <0.3, suggesting a complete loss of binding. Type O and A saliva were from secretors containing H and A antigen, respectively.

### Similar genetic relatedness also exists among GII noroviruses

**Figure 5:**
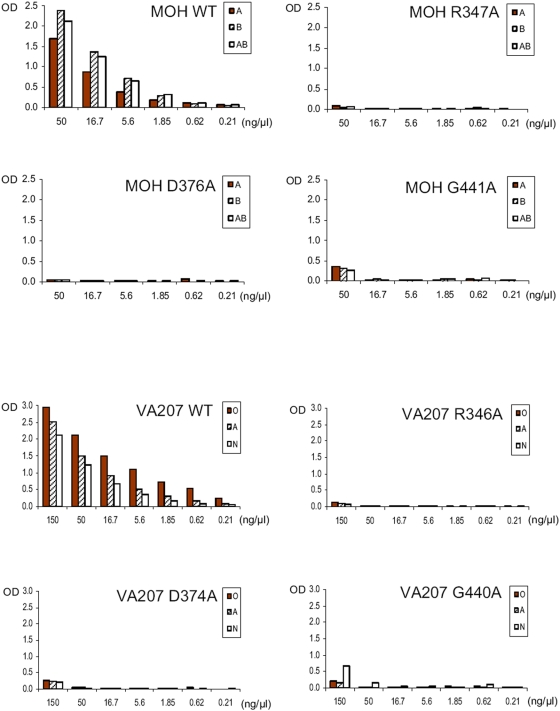
Saliva-based binding results of various mutant P particles of MOH (GII-5, upper panel) and VA207 (GII-9, lower panel) with single amino acid changes at the three GII conserved sites. The X-axes show the protein concentrations of the P particles and the Y axes indicate the optical densities at 450 nm (OD_450_) that were the average value of triplicate experiments. “O”, “A”, “B” and “AB” represent the saliva samples of type O (containing H antigen), A, B, and AB secretor, respectively, while “N” one of nonsecretor.

Similar variations of HBGA binding have also been found among GII strains. For example, strains MOH (GII-5), Buds (GII-2), Parris Island (GII-13), MxV (GII-3), members of the A/B binding group [22], target the common A, B and/or H antigens as VA387 (GII-4) does. Using a similar approach we constructed three mutant P particles (R_347_A, D_376_A and G_441_A) of MOH, each with a single residue change in the three GII conserved sites deduced from VA387 (Fig. 3B) and the binding to HBGAs of all three mutants were completely (R_347_A and D_376_A) or nearly completely (G_441_A) lost (Fig. 5 and Table 4). These results demonstrated that MOH shares the three conserved binding sites with VA387 which is consistent with their recognition of the common A and B antigens.

**Table 4:** Summary of the mutagenesis study of the three sites of the HBGA-binding interface of MOH (GII-5) and VA207 (GII-9) predicted by sequence alignment with that of Norwalk virus (GI) and VA387 (GII).

Mutants of MOH	Binding to type A saliva^1^	Binding to type B saliva^1^	Binding to type AB saliva^1^	Components of the binding interface
WT	+	++	++	
R_347_A	−	−	−	GII-site I
D_376_A	−	−	−	GII-site II
D_349_A	−	−	−	GII-site III

Mutants of VA207	Binding to type O saliva^1^	Binding to type A saliva^1^	Binding to saliva of nonsecretor^1^	Components of the binding interface
WT	+++	++	++	
R_377_A	−	−	−	GII-site I
L_378_A	−	−	−	GII-site II
D_379_A	−	−	−	GII-site III

1 The number of “+” indicated the relative binding affinity of the wild type (WT) and mutant P particles at 16.7 ng/µl to HBGAs: “+++” indicates an OD_450_ >2.0; “++” between 1.0 and 2.0; “+” between 0.3 and 1.0; while “−” indicates an OD_450_ <0.3, suggesting a complete loss of binding. Saliva samples of type A, B, AB, and O were from secretors containing A, B, A/B and H antigen, respectively.

We then examined the role of the three GII conserved sites in HBGA-binding of VA207 (GII-9), a strain of the Lewis binding group that recognizes the Le^x^ and Le^y^ but not the A and B antigens. Construction of three mutants (R_346_A, D_374_A and G_440_A) with a single residue mutation at each of the three binding sites (Fig. 3B) resulted in complete (R_346_A) or nearly complete (D_374_A and G_440_A) loss of binding to HBGAs (Fig. 5 and Table 4). These data showed that VA207 shares the common HBGA-binding interface with those A/B binding strains within GII noroviruses and this has been recently confirmed by the crystal structure of VA207 P dimer in complex with Lewis antigen (Y. Chen, X. Jiang and X. Li to be published data, also see discussion).

## Discussion

**Figure 6:**
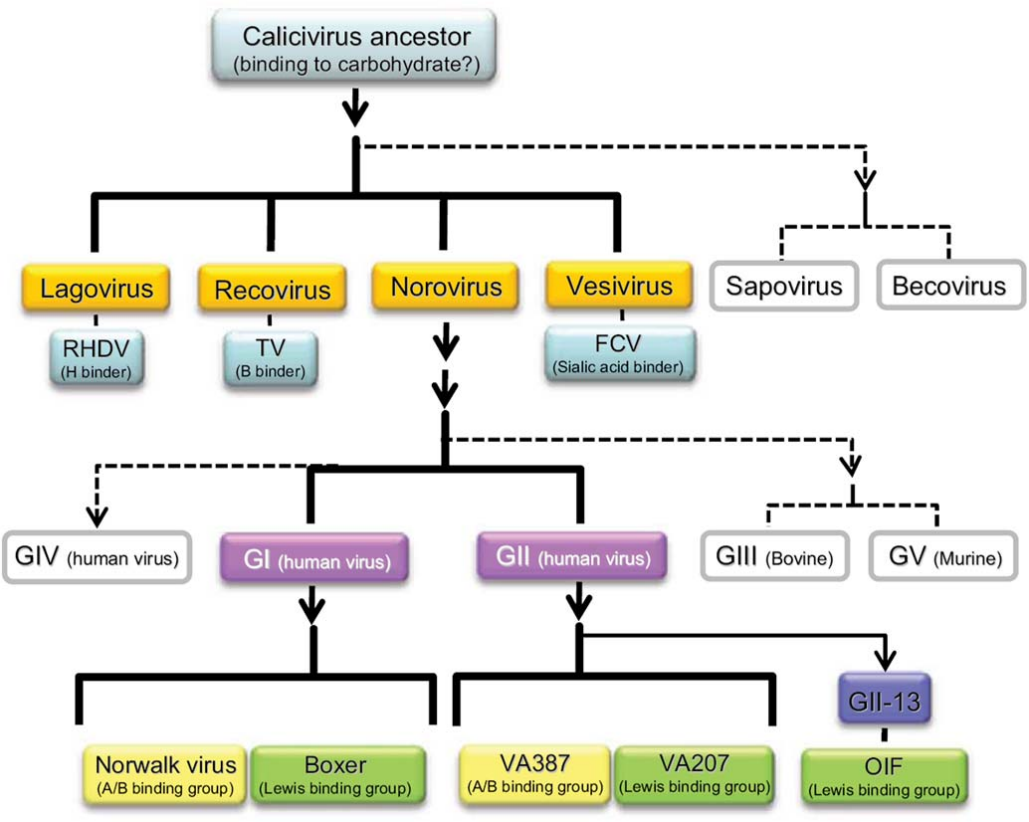
A schematic relationship of the known carbohydrate-binding phenotypes of caliciviruses. Six calicivirus genera may be evolved from a common calicivirus ancestor and at least one strain from four genera (orange) has been shown to bind to carbohydrates. Similarly, five norovirus genogroups (G) may be evolved from a common norovirus ancestor and two of the three human norovirus genogroups have been demonstrated to recognize HBGAs (purple). GI and GII noroviruses share conserved genogroup-specific HBGA-binding interfaces and both genogroups contain strains binding to either A/B/H antigens (A/B binding groups, yellow) or Lewis antigens (Lewis binding group, green). GII-13 (Blue) is a unique genotype that does not share conserved binding sites with other GII genotypes and thus may represent a sublineage parallel to other GII genotypes, in which a strain (OIF) has been shown to bind to Lewis antigens (green). Arrows indicate the direction of evolution. Solid line shows the evolutionary lineages with defined binding to HBGAs, while the dashed line shows the lineages with unknown interaction with carbohydrates. RHDV, rabbit hemorrhagic disease virus; TV, Tulane virus; FCV, feline calicivirus; OIF, norovirus strain that was isolated from troops deployed to the Operation of Iraqi Freedom.

In this study, we used sequence alignment, structural analysis and site-directed mutagenesis to examine the evolutionary relatedness of human noroviruses in terms of their interaction with the HBGA receptors. We showed that strains with distinct HBGA binding patterns within genogroups share common receptor binding interfaces in their interactions with variable HBGAs, likely tuned up by subtle structural differences within the binding interfaces. At the same time, strains in different genogroups that use different binding interfaces, as defined by their locations and sequence motifs, can recognize the same HBGA-targets, pointing to the overall functional and structural similarity of these distinct binding sites. These results provide evidence that the human HBGAs exert an important selection pressure in norovirus evolution. The two major genogroups (G I and GII) of human noroviruses that cause acute gastroenteritis represent two major evolutionary lineages, while strains in the A/B and Lewis binding groups within the two genogroups, such as those represented by the Norwalk virus and Boxer in GI and those by VA387 and VA207 in GII, may further divide into evolutionary sub-lineages as a result of divergent evolution within each branch (Fig. 6).

The HBGA-binding interfaces of the two major genogroups of human noroviruses share some similarity in the overall structure and location; both are located in the outermost P2 regions of the capsids [13], [14], [15] and both are composed of three major structural components, corresponding to the bottom and the walls of the binding pocket (Fig. 1). However, the two binding interfaces differ in their primary sequences, detailed locations, and modes of interaction with the HBGA-receptors [13], [14], [15]. The binding interface of the GI strains (Norwalk virus) is constituted by three groups of amino acids from the P2 subdomain and positioned mainly in one P monomer, although it is near the interface of two P monomers of the Norwalk virus P dimer. On the other hand, the binding interface of the GII viruses (VA387) is composed of residues from both P1 and P2 subdomains and is located right at the interface of two monomers in the VA387 P dimer [13], [14], [15], [35]. The conservation of the binding interfaces within GII has been confirmed by the crystal structures of the VA207 P dimers in complex with the Lewis x and Lewis y tetrasaccharides, respectively, in which the binding interface of VA207, a Lewis binding strain, is constituted by the conserved amino acids and interacts with the α-1,3/4 fucose of the Lewis y antigen in a similar way like that of VA387 (Y. Chen, X. Jiang and X. Li, to be published data).

The two types of binding interfaces differ also in their binding modes to HBGAs. Based on crystal structures of the P dimers complexed with oligosaccharides, Norwalk virus has a smaller or narrower binding interface, while VA387 has a larger or broader one (Fig. 1, [13], [14], [15]). As a result only two sugars of the A trisaccharide and the H pentasaccharide are involved in interaction with Norwalk virus [13], [15], as opposed to all three sugars of the A and B trisaccharides in case of VA387 [14], [35]. In addition, more amino acid residues of VA387 appear to be involved in binding to HBGAs, compared to Norwalk virus. Specifically, crystal structures revealed 11 residues of VA387 P domain interacting with the B trisaccharides, as opposed to only 7 in case of Norwalk virus P domain binding to the A or H oligosaccharides [13], [14], [15]. Furthermore, mutagenesis studies mapped another 8 amino acids around the binding interface of VA387 affecting the binding function [35], while only 2 such residues of Norwalk virus were found (this report). Nevertheless, the aforementioned binding modes are based solely on the P dimers interacting with oligosaccharides under the condition of co-crystallization. The native interactions between norovirus and HBGAs *in vivo* remain to be elucidated.

Another observation emerging from this study is the possibility of interplay between convergent and divergent evolution of noroviruses. The two major genogroups (GI and GII) of human noroviruses are characterized by distinct genetic traits with significant differences in the primary sequence within their P domains. These two distinct lineages may have evolved in the course of divergent evolution from a common ancestor. On the other hand, the acquisition of the common function of binding to HBGAs by distinct binding interfaces and modes is consistent with functional convergence as a result of adaptation to and selection by the same niche of human HBGAs. The two strains described in this study, VA387 and Norwalk virus, provide strong support for this hypothesis. Convergent evolution of protein function and/or structure in conjunction with acquired ligand binding specificity has been observed previously [37], [38], [39]. One such example includes sugar binding families of LacI/GalR repressors and their PBP analogues, in which evolutionarily divergent lineages acquired independently similar ligand binding patterns through convergent evolution [40].

The fact that almost all known HBGAs have their noroviral counterparts suggests that noroviruses are highly adaptive human pathogens. In addition, it has been noted that some strains with conserved binding interfaces appear not to recognize HBGAs, such as the Desert Shield virus (DSV, GI-3) [22] and Hunter virus (GII-4) [41], while other strains lacking the conserved binding interfaces retain the HBGA-binding ability, such as OIF of the GII-13 noroviruses [22], [42]. These variations further highlight the adaptive nature of noroviruses that may recognize other carbohydrates or even non-carbohydrates as receptors. As long as noroviruses remain a human pathogen, the diversity of HBGA-binding patterns seen today will probably extend into the future.

Limited studies have shown that the GI and GII noroviruses are biologically different. For example, the GI noroviruses are more involved in environmental contamination and cause outbreaks year around without apparent seasonal peaks, while GII strains are easier to spread via person-to-person contact [9], [10], [11] and commonly cause outbreaks with clear fall/winter peaks. While future studies are required to identify factors and genetic markers responsible for these differences, this work can help to elucidate the evolutionary relatedness of the GI and GII noroviruses and improve the classification of caliciviruses (Figure 6). Each of the four major genera and the two newly discovered “Becovirus” [3] and “Recovirus” [2] genera should represent an evolutionary lineage in this virus family. While each of them has adapted well into individual host species, the binding to carbohydrates has apparently been maintained or acquired in at least some strains of most genera of caliciviruses, other than human noroviruses. For example, the rabbit hemorrhagic disease virus (RHDV) of the *Lagovirus* and the Tulane virus (TV) of the *Recovirus* recognize HBGAs [33], [34], while feline calicivirus (FCV) bind to sialic acid [43]. Since the common ancestor of these genetically distinct species might not possess the HBGA binding trait, one might speculate that these common characteristics were acquired independently as a result of adapting to similar biological niches, suggesting a possible convergent evolution of caliciviruses.

Our mutagenesis study further demonstrated that, in addition to the conserved binding sites, a number of nearby amino acids also play an important role in the binding specificity to HBGAs, possibly by contributing to the conformational flexibility of the carbohydrate binding interfaces, and these residues are less conserved. For example, residues Q_331_, K_348_, I_389_, and G_392_ of VA387 are likely involved in the binding to the A but not the B antigens [35], while S_338_, A_430_ and Y_431_ of Norwalk virus affect the binding strongly to H but weakly to A antigen (this study). Similar role of D393 of another GII-4 strain was also observed [41]. The recent studies on the globally dominant GII-4 noroviruses suggests that the host herd immunity may play a role in the epochal evolution of GII-4 viruses [41], [44]. Future studies focusing on these non-conserved residues for their potential roles in the antigenicity and immunogenicity of the viruses may be necessary.

In this study 39 mutant P particles of four strains (Norwalk, Boxer, MOH, and VA207) have been generated to address the conservation issue of the HBGA binding interfaces of noroviruses. This task would be very difficult to complete by using the VLPs as the model, because VLP production are very time-consuming compared to P particles. In our previous studies we have demonstrated that the P particle is a good model for studying norovirus-HBGAs interaction by the observations that P particle uses the same HBGA binding interface and shares very similar HBGA binding profile as that of its VLP counterpart [17], [18], [35], [36]. In addition, we used the saliva binding assay for its simplicity, convenience and sensitivity. All saliva samples used in this report have been well characterized for their phenotypes and binding patterns to noroviruses in our previous studies [17], [18], [21], [22], [35], [36], [45]. We do not expect significant differences with respect to synthetic oligosaccharide-based assays in evaluation of the importance of HBGA binding sites.

The findings of the conservation of HBGA-binding interfaces within genogroups can greatly facilitate the design and development of therapeutics against noroviruses. For example, a single compound that inhibits the function of the conserved HBGA-binding interface may be capable of blocking infection of all strains with the same type of HBGA binding interface. Thus, only two compounds might be sufficient to block most noroviruses in the two genogroups studied here, each group sharing a similar binding interface that could be blocked by one common inhibitor.
